# A study to assess changes in myocardial perfusion after treatment with spinal cord stimulation and percutaneous myocardial laser revascularisation; data from a randomised trial

**DOI:** 10.1186/1745-6215-9-9

**Published:** 2008-02-28

**Authors:** Sadia N Khan, Duncan C McNab, Linda D Sharples, Carol J Freeman, Ian Hardy, David L Stone, Peter M Schofield

**Affiliations:** 1 Department of Cardiology, Papworth Hospital, Papworth Everard, Cambridge, UK; 2 MRC Biostatistics Unit, Robinson Way, Cambridge, UK; 3 Department of Research and Development, Papworth Hospital, Papworth Everard, Cambridge, UK; 4 Department of Anaesthetics, Papworth Hospital, Papworth Everard, Cambridge, UK

## Abstract

**Background:**

Spinal cord stimulation (SCS) and percutaneous myocardial laser revascularisation (PMR) are treatment modalities used to treat refractory angina pectoris, with the major aim of such treatment being the relief of disabling symptoms. This study compared the change in myocardial perfusion following SCS and PMR treatment.

**Methods:**

Subjects with Canadian Cardiovascular Society class 3/4 angina and reversible perfusion defects as assessed by single-photon emission computed tomographic myocardial perfusion scintigraphy were randomised to SCS (34) or PMR (34). 28 subjects in each group underwent repeat myocardial perfusion imaging 12 months post intervention. Visual scoring of perfusion images was performed using a 20-segment model and a scale of 0 to 4.

**Results:**

The mean (standard deviation) baseline summed rest score (SRS) and stress scores (SSS) were 4.6 (5.7) and 13.6 (9.0) in the PMR group and 6.1 (7.4) and 16.8 (11.6) in the SCS group. At 12 months, SRS was 5.5 (6.0) and SSS 15.3 (11.3) in the PMR group and 6.9 (8.2) and 15.1 (10.9) in the SCS group. There was no significant difference between the two treatment groups adjusted for baseline (p = 1.0 for SRS, p = 0.29 for SSS).

**Conclusion:**

There was no significant difference in myocardial perfusion one year post treatment with SCS or PMR.

## Introduction

The SPiRiT trial is an open label, single-centre, parallel group randomised trial comparing percutaneous myocardial laser revascularisation (PMR) with spinal cord stimulation (SCS) in patients with refractory angina pectoris [1]. These techniques have been shown to improve symptom control [2-6] in this group, although there is debate as to the mechanisms underlying the clinical response [7-10]. In accordance with the previous studies on laser revascularisation carried out at this institution [2,3,11] and with the recommendations of the European Society of Cardiology Joint Study Group [12], the presence of a reversible perfusion defect was an inclusion criterion for this study. Perfusion imaging was repeated 12 months post intervention in order to determine whether SCS and PMR treatment lead to an improvement in perfusion, a possible mechanism of action of these therapeutic modalities, and whether such change correlated with change in angina score as measured by CCS class.

## Methods

The inclusion/exclusion criteria and methods of the SPiRiT trial have been previously reported [1]. Briefly, this open label, single centre, parallel group randomised controlled trial was carried out in a tertiary referral centre for patients with cardiovascular disease. The main inclusion criteria were limiting angina despite maximally tolerated antianginal medication, angiographically documented coronary disease unsuitable for conventional revascularisation (this judgement was made by a Consultant Interventional Cardiologist in conjunction with the referring Consultant Cardiologist/Cardiothoracic surgeon) and reversible ischaemia on Tc-99m sestamibi scanning. Exclusion criteria included myocardial wall thickness < 8 mm in the areas to be treated by PMR, implanted pacemakers or defibrillators or comorbidity that was considered by the assessing clinician to be of greater significance than angina pectoris. Ethical approval was obtained from Huntingdon Local Research Ethics Committee (reference H00/557) prior to study commencement and all subjects gave informed consent in accordance with the Declaration of Helsinki.

68 subjects were randomised, of whom 34 were treated with SCS and 34 with PMR. Perfusion scanning was performed at baseline and 12 months post intervention. A 2-day protocol was followed. The rest scan was performed with 400 MBq of Tc-99m sestamibi being injected into a peripheral vein whilst the patient was in a fasted state. Pharmacological stress was performed with adenosine at an infusion rate of 140 mcg/kg/min for 6 minutes used as the stress agent in more than 90% of cases. The same dose of Tc-99m sestamibi was administered 3 minutes into the infusion. Dobutamine was used as an alternative stress agent in subjects with significant reversible airways disease. The same stress agent was used for the pre- and post-treatment scans for each individual patient. All scans were performed a minimum of one hour after injection after a fatty meal. A single-headed Elscint Apex SP4 gamma camera with a low energy collimator, a 64 × 64 matrix and a 6.9 mm pixel size (Elscint Ltd, Haifa, Israel) was used. Acquisitions were obtained in step and shoot mode, with 64 projections over a 180-degree circular orbit, with a three-degree increment, a matrix size of 64 × 64 and 18 seconds/projection. The studies were reconstructed using filtered backprojection without attenuation or scatter correction and realigned along the heart axis.

### Scintigraphic image analysis

**Figure 1:**
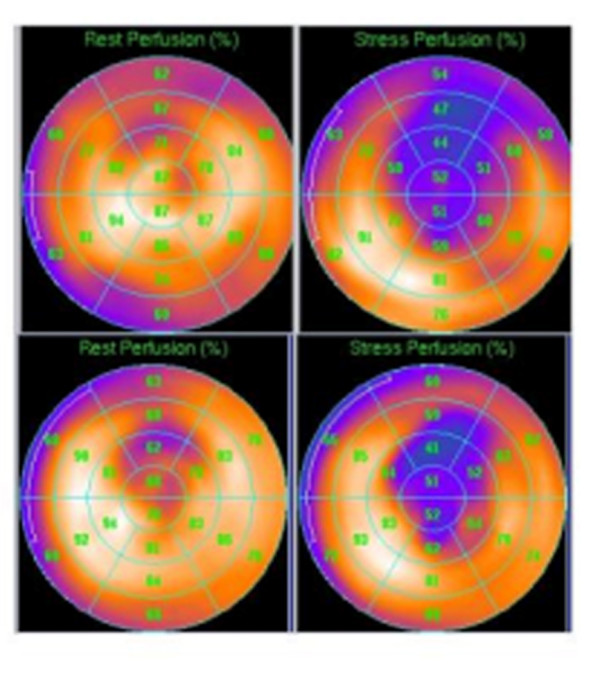
Example Scintigraphic Rest and Stress Images pre (upper panel) and 1-year post treatment (lower panel) with PMR to the anterior wall.

For perfusion analysis the left ventricle was divided into 20 segments, with 3 radial layers of 6 segments and two apical segments. All images were visually assessed by consensus of 2 experienced observers blinded to clinical data. Perfusion was scored semiquantitatively using a 5-point scale (0 = normal uptake; 1 = equivocal uptake; 2 = moderately reduced uptake; 3 = severely reduced uptake and 4 = no uptake) [13,14]. The summed stress score (SSS) and the summed rest score (SRS) were defined as the sum of all the scores on the stress and rest images respectively with the summed difference score (SDS) being the difference between the SSS and the SRS. These scores were calculated for each patient at baseline and at 1-year follow-up. An example from a subject treated with PMR is shown in Figure 1.

### Statistical analysis

The CCS class was compared using the Mantel-Haenszel test with class as a linear trend. Significant improvement in CCS was defined as at least a 2-class decrease and the proportion of patients who had a significant improvement in each group was compared using Fisher's exact test. The SSS, SRS and SDS were summarised as the mean and standard deviation for each group. In order to assess the effect of treatment on these scores generalised linear models with normal link were used with baseline score and group as independent variables and one-year score as the dependent variable. The treatment effect and 95% confidence intervals from these models are reported. Since the distributions of the scores were not completely symmetrical these results were re-assessed against the results of Mann-Whitney U tests, comparing the two groups for differences between baseline and one year.

## Results

### Patient characteristics

Baseline and follow-up imaging was available in 56 patients (28 in each group). The commonest reasons for failure to complete follow-up scanning were withdrawal from the study or patient refusal. The baseline characteristics are as shown in table 1. Analysis of the baseline studies from the 12 patients who did not undergo repeat scanning at one year did not suggest that these patients differed significantly from the group as a whole. Antianginal medication did not change during the period of follow-up.

**Table 1:** Baseline Characteristics

	PMR (n = 28)	SCS (n = 28)
Mean age in years (SD)	62.3 (9.7)	64.4 (7.5)
Previous revascularisation	28 (100%)	28 (100%)
Baseline medication		
Beta-blockers	22 (79%)	23 (82%)
Calcium channel blockers	22 (79%)	23 (82%)
Aspirin	24 (86%)	26 (93%)
Nicorandil	26 (93%)	24 (86%)
Long-acting nitrates	23 (82%)	21 (75%)
ACE -inhibitors	13 (46%)	14 (50%)

Follow-up medication		
Beta-blockers	20 (71%)	24 (86%)
Calcium channel blockers	20 (71%)	23 (82%)
Aspirin	23 (82%)	23 (82%)
Nicorandil	25 (89%)	22 (79%)
Long-acting nitrates	23 (82%)	22 (79%)
ACE – inhibitors	13 (46%)	15 (54 %)

At baseline, all patients were in CCS class 3 or 4. Nineteen (68%) of PMR and 17 (61%) of SCS patients were in class 3 (p = 0.781). At one year more SCS patients were in CCS class 1 or 2 (Table 2) and the difference was marginally significant at the traditional level (p = 0.059).

**Table 2:** CCS Class at year one

CCS class at 1 year	PMR (n = 28)	SCS (n = 28)
1	1 (4%)	6 (21%)
2	12 (43%)	13 (46%)
3	7 (25%)	4 (14%)
4	8 (29%)	5 (18%)

Four PMR patients had a 2-class improvement in CCS compared to nine SCS patients who had a 2 class and 2 who had a 3 -class improvement (Table 3). Again, the greater proportion of patients with a significant improvement in CCS class in the SCS group was close to significant at traditional levels (p = 0.068).

**Table 3:** Change in CCS Class

Change in CCS at 1 year	PMR (n = 28)	SCS (n = 28)
-3	0 (0%)	2 (7%)
-2	4 (14%)	9 (32%)
-1	11 (39%)	9 (32%)
0	9 (32%)	6 (21%)
1	4 (14%)	2 (7%)

### Perfusion results

Summed scores were available for 28 PMR and 28 SCS patients at both baseline and one year after treatment. The data are summarised in table 4. Adjusting for baseline there was no significant difference between treatment groups at one year in SRS (-0.004 (95%CI: -2.1, 2.1), p = 1.00). For the SSS the SCS group was 2.4 lower than the PMR group after adjusting for baseline but this did not reach significance (-2.4 (95% CI: -6.8, 2.0), p = 0.29). Similarly, for the SDS the SCS group was 2.4 lower than the PMR group and again this did not reach statistical significance (-2.4 (95%CI: -6.2, 1.3), p = 0.21). The Mann-Whitney U test gave similar results for all 3 measures.

**Table 4:** Mean and standard deviation for summed scores by treatment group

Variable	PMR group (n = 28)	SCS group (n = 28)
**Baseline**		

Summed rest score	4.6 (5.7)	6.1 (7.4)
Summed stress score	13.6 (9.0)	16.8 (11.6)
Summed difference score	9.0 (8.4)	10.6 (10.2)

**One year**		

Summed rest score	5.5 (6.0)	6.9 (8.2)
Summed stress score	15.3 (11.3)	15.1 (10.9)
Summed difference score	9.8 (8.7)	8.3 (9.1)

### Adverse Events

Sixty-seven non-fatal adverse events were recorded in the first year for the 56 patients with baseline and one year imaging (Table 5). The SCS group reported significantly more adverse events than the PMR group (p < 0.001). Forty-nine events occurred in 17 patients in the SCS group, with 22 events categorised as being related to the SCS procedure. Eleven patients in the PMR group reported 18 adverse events. Three events were related to the PMR procedure, one of which occurred in a patient randomised to SCS. Of the 42 events not related to the procedure 36 were related to the underlying disease and the difference between the groups was not significant (p = 0.126).

**Table 5:** Adverse Events reported in the first year

	SCS	PMR
Disease related		

Unstable angina	16	9
Myocardial infarction	2	0
Worsening angina	6	3
Total disease related	24	12
SCS related		
Infection of SCS system	0	NA
Undesirable change in stimulation	14	NA
Pain at neurostimulator site	5	NA
Neurostimulator migration	2	NA
Lead migration	1	NA
PMR related		
Pseudoaneurysm	0	1
Groin haematoma	1	1

Other miscellaneous	2	4

TOTAL EVENTS	49	18

Total Events excluding SCS/PMR related	26	16

## Discussion

Refractory angina pectoris is an increasingly common clinical problem with significant associated morbidity. Many different treatment modalities have been reported to alleviate symptoms and hence improve quality of life for patients with this condition. When assessing the efficacy of such therapies it is important to acknowledge that because angina is a symptom, it is subjective in nature. This is particularly so in the situation of medical devices, where a significant placebo effect is known to exist [3,4]. Consequently, caution must be exercised in the interpretation of studies based solely upon anginal symptoms, or other subjective endpoints such as hospital admissions, and exercise performance, although from the individual patient's point of view, these endpoints may be the most important.

Previous data assessing changes in perfusion following both SCS and PMR has been inconclusive. Three PET studies of myocardial perfusion and SCS in humans have been published. Two studies did not demonstrate any increase in the primary outcome measure, total myocardial blood flow following SCS. [15-17] The third study, which only assessed resting flow, reported an increase in total myocardial blood flow. Two of the studies, as part of a secondary analysis, reported "homogenisation of flow" i.e. when comparing regional flow after SCS to baseline results, blood flow increased in regions of low baseline flow and fell in regions of high baseline flow. This flow homogenisation, which was suggested to underpin the beneficial effects of SCS could also be explained by the phenomenon of regression to the mean. Diedrichs et al.[18] reported an improvement in myocardial perfusion in some patients 12 months after treatment with SCS. However, this improvement, unlike improvements in symptom severity, functional capacity and quality of life, was not demonstrated at 3 months rasing the possibility that the perfusion changes were secondary to improved functional capacity. Similarly, following PMR treatment, most clinical studies have failed to show a consistent change in myocardial perfusion to correspond to improvements recorded in angina class and exercise capacity, although investigators have reported improvements in myocardial perfusion some using different laser systems to that used here [8,10,19-21]. Some of these differences may be explained by patient and laser system selection. The laser used in this study was the Cardiogenesis system which differs from the Biosense DMR[21] system in that angiographic and perfusion data are used to identify areas to be treated by laser as opposed to electromechanical mapping of the left ventricular cavity, and in that the DMR laser is a 'contact' system such that the depth of the channels created is significantly less.

This is the first study to compare change in perfusion in refractory angina for two active treatments. There was no significant difference in perfusion between or within treatment groups at year one. There are several limitations of this study and these may account for this negative finding. Although it is known that the symptoms of refractory angina pectoris remain remarkably constant in most patients [22], it is clear that perfusion defects can vary considerably, independently of study processing variables [23]. In addition, perfusion defects were assessed using adenosine as a selective vasodilator with sestamibi as the tracer agent. Patients with refractory angina often have diffuse coronary artery disease with impaired fractional flow reserve hence it may not be possible to demonstrate a change in perfusion, even in the presence of significant ischaemia. Also, this study was relatively small and was not powered for this secondary outcome measure.

## Conclusion

In conclusion, this study has not demonstrated a significant change in perfusion one -year post treatment. We would recommend that studies of refractory angina therapies include objective measures of myocardial performance and that consideration be given to techniques other than myocardial perfusion imaging using adenosine as a stressor.

## Abbreviations

SCS: spinal cord stimulation, PMR: percutaneous myocardial laser revascularisation, PET: positron emission tomography, SRS: summed rest score, SSS: summed stress score, SDS: summed difference score.

## Competing interests

This study was sponsored by Medtronic SA, who were responsible for funding of the trial related investigations such as perfusion scans and treadmill tests, research staff for data collection and travelling expenses for the subjects. The sponsor had no role in study design, data collection and interpretation or in the decision to submit the report for publication.

## Authors' contributions

SK carried out perfusion image and data analysis and drafted the manuscript. DM was responsible for patient management, performed SCS and PMR procedures and helped with perfusion data analysis. LDS performed the data analysis, provided statistical support and drafted the manuscript. CJF was responsible for study management, assisted with data analysis and drafting of the report. DLS was responsible for perfusion data acquisition and analysis. IH assisted with patient management particularly SCS system implantation. PMS was responsible for data analysis, patient management, writing of the report and acts as guarantor and chief investigator for this study. All authors read and approved the final manuscript.
